# Microscale advection governs microbial growth and oxygen consumption in macroporous aggregates

**DOI:** 10.1128/msphere.00185-24

**Published:** 2024-03-26

**Authors:** Rachel Shen, Benedict Borer, Davide Ciccarese, M. Mehdi Salek, Andrew R. Babbin

**Affiliations:** 1 Department of Earth, Atmospheric and Planetary Sciences, Massachusetts Institute of Technology, Cambridge, Massachusetts, USA; 2 Department of Biological Engineering, Massachusetts Institute of Technology, Cambridge, Massachusetts, USA; University of Wisconsin-Madison, Madison, Wisconsin, USA

**Keywords:** mass transport, biofilm establishment, microbial aggregates, diffusion limitation, advective mass-transport

## Abstract

Most microbial life on Earth is found in localized microenvironments that collectively exert a crucial role in maintaining ecosystem health and influencing global biogeochemical cycles. In many habitats such as biofilms in aquatic systems, bacterial flocs in activated sludge, periphyton mats, or particles sinking in the ocean, these microenvironments experience sporadic or continuous flow. Depending on their microscale structure, pores and channels through the microenvironments permit localized flow that shifts the relative importance of diffusive and advective mass transport. How this flow alters nutrient supply, facilitates waste removal, drives the emergence of different microbial niches, and impacts the overall function of the microenvironments remains unclear. Here, we quantify how pores through microenvironments that permit flow can elevate nutrient supply to the resident bacterial community using a microfluidic experimental system and gain further insights from coupled population-based and computational fluid dynamics simulations. We find that the microscale structure determines the relative contribution of advection vs diffusion, and even a modest flow through a pore in the range of 10 µm s^−1^ can increase the carrying capacity of a microenvironment by 10%. Recognizing the fundamental role that microbial hotspots play in the Earth system, developing frameworks that predict how their heterogeneous morphology and potential interstitial flows change microbial function and collectively alter global scale fluxes is critical.

**IMPORTANCE:**

Microbial life is a key driver of global biogeochemical cycles. Similar to the distribution of humans on Earth, they are often not homogeneously distributed in nature but occur in dense clusters that resemble microbial cities. Within and around these clusters, diffusion is often assumed as the sole mass-transfer process that dictates nutrient supply and waste removal. In many natural and engineered systems such as biofilms in aquatic environments, aggregates in bioremediation, or flocs in wastewater treatment plants, these clusters are exposed to flow that elevates mass transfer, a process that is often overlooked. In this study, we show that advective fluxes can increase the local growth of bacteria in a single microenvironment by up to 50% and shape their metabolism by disrupting localized anoxia or supplying nutrients at different rates. Collectively, advection-enhanced mass transport may thus regulate important biogeochemical transformations in both natural and engineered environments.

## INTRODUCTION

Microbial life in natural and engineered environments most often exists in discretely localized microenvironments or hotspots such as biofilms, aggregates, flocs, particles, and granules to name a few (1). Unifying these environments is the principle of living in confinement, whereby the collective activity of the community creates steep nutrient and oxygen gradients within and around these environments. In turn, the resulting diffusive mass transport dictates nutrient acquisition of individual cells and gives rise to a landscape of different metabolisms, all across a spatial scale of a few micrometers. These self-engineered microenvironments are not isolated occurrences in specific habitats but are abundant and ubiquitous, and collectively play a crucial role in driving global biogeochemistry (2). For instance, soil bacterial hotspots, such as aggregates and the rhizosphere (3), are important sources of greenhouse gases (4) due to the localized development of anoxic niches (5). In the oceans, similar anoxic microenvironments are observed in settling marine snow (6, 7) that support anaerobic metabolisms (8) and can nearly double the niche size for marine denitrification on a global scale (9). The occurrence of anoxic microenvironments due to diffusive limitations is not exclusive to natural environments. They also emerge in systems that are optimized for oxygen exchange such as flocs in activated sludge (10) or within pulmonary biofilms of cystic fibrosis patients (11). At finer scales within a microenvironment, the steep nutrient gradients govern the spatial organization of the bacterial community (12, 13) whereas even a single species shows phenotypic heterogeneity depending on the precise location of the cells inside the hotspot (13–15). This diffusive limitation not only generates anoxic conditions but also affects all resources when localized consumption exceeds supply.

Yet, typically overlooked is the role of advection in addition to diffusion in setting the chemical landscape where flow around microbial assemblages can impact the way organisms perceive nutrient gradients and alter nutrient fluxes (16). For instance, the degradation rate of alginate particles increases due to enhanced mass fluxes across the particle as a function of flow velocity (17). These relationships between the localized flow and the increase in mass transport due to bulk advection are primarily studied for simplified geometries such as solid spheres or cylinders. Natural microbial hotspots that encounter flow such as marine particles (18), biofilms in streams and intertidal zones (19–21), or flocs in activated sludge (22) typically manifest irregular shapes and structures that may support interstitial flow through pores or gaps between cell clusters. The relative importance of advective vs diffusive nutrient transport in these systems is then a function of the diffusivity of the nutrient, the localized flow velocity, and the spatial scale (16). Mathematically, this is captured by the dimensionless Péclet number (Pe), defined as the ratio between the rate of advective and diffusive mass transport (Eq. 1), where *L* is the spatial scale (e.g., the diameter of the cylinder or sphere), *v* the flow velocity, and *D* the diffusivity of the considered nutrient.


Eq. 1 $$Pe=\;\frac{Lv}{D}$$

At Pe smaller than unity, nutrients are primarily transported by molecular diffusion, whereas advective transport dominates at high Pe. Since diffusive fluxes are restricted to short distances, a landscape of Pe emerges at the microscale (23) with regions of high advective mass transport due to localized fluid flow and regions where mass transport is restricted to diffusive fluxes. For small molecules (diffusivities on the order of 10^−9^ m^2^ s^−1^), a pore radius of 50 µm and flow velocity of 10 µm s^−1^ result in a Pe of 1, dimensions which are relevant to many microbial hotspots. This suggests that hotspots may often experience tipping points between diffusion- and advection-dominated nutrient supply. Recently, the impact of the microscale structure on the localized flow around marine particles has been investigated (24), but the overall effect of the microenvironment morphology on nutrient supply, microbial growth conditions within the porous structure, and the carrying capacity of individual microenvironments remain unclear.

**Fig 1:**
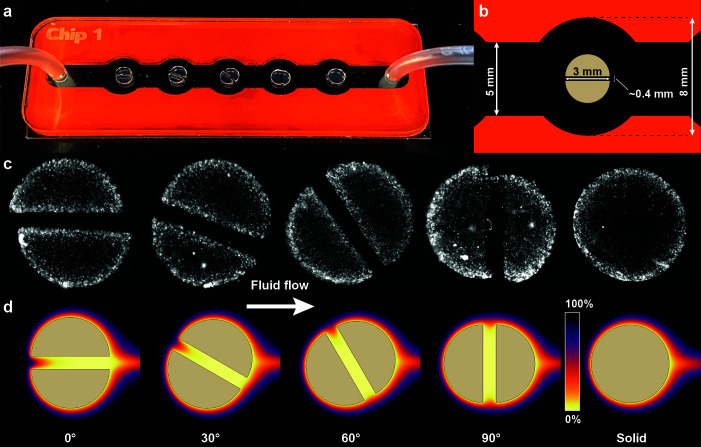
Visualization of the experimental system and predicted oxygen concentrations. (**a**) Photograph of the experimental system highlighting the flow chamber and hydrogel discs with different angular orientations of the channels. (**b**) Dimensions of the flow chamber and discs. (**c**) Micrograph showing bacterial colonies throughout the hydrogel discs after 24 h incubation. Brighter areas correspond to higher bacterial density. The contrast in these images has been increased evenly to highlight bacterial colonies. (**d**) Spatial oxygen saturation around the hydrogel discs after 24 h predicted with COMSOL.

In the present study, we aim to understand how advective flows through a microenvironment alter the spatial growth conditions, and how these alterations affect the overall carrying capacity of the microbial hotspots. We hypothesize that large pores that support interstitial flow can critically elevate nutrient supply to the microenvironment with significant consequences for microbial growth and community function. We quantify the relationship between flow through microenvironments, the elevated nutrient supply, and the proliferation of the resident bacterial population by observing the growth of bacterial colonies embedded in agarose discs subjected to flow in millifluidic devices (Fig. 1a). Here and throughout the manuscript, we refer to the agarose gel particles embedded within the microfluidic devices as discs. We further use the term macropores for pores that permit advective flow and micropores for the small-scale pore structure within a gel matrix (such as agarose) that restricts mass transport to diffusion. We control the bulk flow velocity around 3 mm diameter agarose discs and create a channel through the discs of approximately 400 µm width to imitate a large pore (Fig. 1b). The contribution of advective fluxes (via laminar flow through the macropores) to the total nutrient supply is controlled by changing the angle of this channel (0°, 30°, 60°, and 90°) relative to the flow direction (Fig. 1c and d, see Materials and methods section for details on the device and disc fabrication).

**Fig 2:**
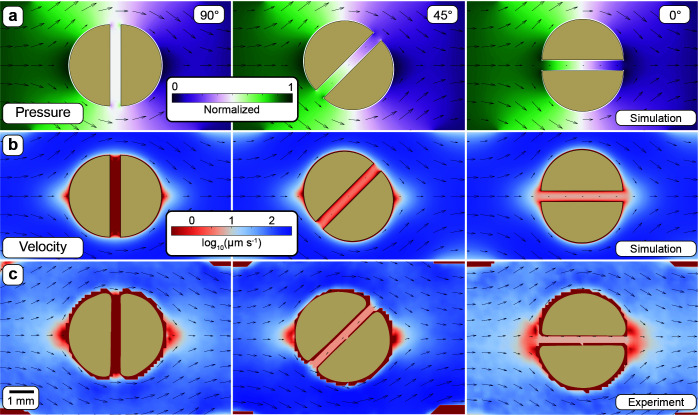
Resulting velocity field around and through the discs as a function of the channel angle. (**a**) Distribution of pressure and flow velocity around and within a cylinder containing a channel. The orientation of the channel relative to the bulk flow direction determines the pressure gradient along the channel axis (visible as the change in color across the channel). The pressure is normalized as it varies with the location of the particle inside the flow chamber. (**b**) Visualization of the predicted flow field around discs with the same channel angle using COMSOL. If the channel is perpendicular to the flow (90°), there is no pressure difference across the channel, and the resulting net flow through the channel is thus 0. A channel at an angle of 45° to the approaching flow results in a slower flow through the channel. For a channel in line with the flow direction (0°), the stagnation point upstream of the disc (the location where the streamline directly intercepts the disc) disappears since the approaching flow passes through the channel. (**c**) Visualization of the flow field around the discs using particle image velocimetry (PIV).

Laminar flow, a flow regime that is characterized by discrete water parcels following smooth paths and the absence of turbulent mixing, around a cylinder or sphere results in a gradient of pressure (relative to the bulk fluid pressure) across the cylinder or sphere (Fig. 2a). The resulting pressure gradient along the axis of the channel accelerates the fluid. Within the channel, fluid that interfaces with the channel walls has a velocity of 0 (no-slip assumption). Attraction between individual water molecules creates drag between adjacent fluid layers which results in frictional deceleration of the fluid. This frictional resistance is proportional to the flow velocity in the channel and inversely proportional to the channel size (i.e., narrower channels result in higher friction and lower flow velocity compared to wider channels exposed to the same pressure gradient). The steady-state flow velocity inside the channel is thus determined when acceleration due to the pressure gradient force equals deceleration due to frictional resistance. The orientation of the channel inside the cylinder governs the pressure gradient across the channel due to the difference in pressure around the disc. A channel that is in line with the flow results in the largest pressure gradient (and thus the highest flow through the channel), whereas a channel perpendicular to the flow results in a negligible pressure gradient (and thus no corresponding flow). A spatial visualization of the flow magnitude and direction predicted in COMSOL and validated experimentally using particle image velocimetry (PIV) is shown in Fig. 2b and c, respectively. In summary, the flow through a channel is determined by the bulk fluid velocity, channel width, and orientation relative to the bulk flow direction which we strategically vary in our experimental system and numerical simulations.

The objectives of this study are (i) to quantify the influence of advective nutrient supply at the microscale on the growth of bacterial populations inside gelatinous microenvironments exposed to flow and (ii) to generalize these insights using simple engineering and fluid mechanics principles that transcend phenomenological-specific examples. Understanding the bidirectional interaction between bacterial populations and their heterogeneous microenvironment is fundamental to quantifying the influence of such microenvironments on global-scale biogeochemical fluxes and developing predictive capabilities for engineering applications such as in wastewater treatment plants or bioremediation.

## RESULTS

### Bacterial carrying capacity as a function of interstitial flow

**Fig 3:**
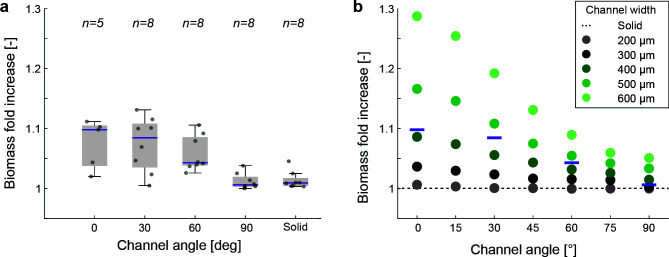
Relative biomass increase as a function of channel angle. (**a**) Relative biomass increase as a function of the channel angle where *n* is the number of experimental replicates. The relative increase is calculated as the increase in biomass when compared to a solid disc as described in the Materials and Methods section. (**b**) Relative biomasses increase as a function of the channel angle and channel widths when simulated using COMSOL. Purple lines are the mean relative biomass increase from the experimental data with an approximate channel width of 400 µm.

There is a significant influence of the channel angle (as a proxy for increased nutrient flux) on the relative biomass increase (Fig. 3a, Welch ANOVA, *P*-value = 0.0012) with the highest relative increase for the in-line channel (0° angle, mean ± std relative increase of 7.52% ± 4.08%). This influence decreases with increasing channel angle where a perpendicular channel to the flow (90° angle, mean ± std relative increase of 1.18% ± 1.33%) is indistinguishable from a solid disc (mean ± std relative increase of 1.38% ± 1.45%). Solid discs show a slight relative biomass increase due to outliers (i.e., individual large colonies deep inside of the discs that are outliers when compared to the general trend, Fig. S1 and S2; Table S1). To gain insights into how the microscale geometry and associated advective fluxes drive bacterial growth, we adopted a digital twin model created in the multiphysics modeling software COMSOL (details of the model can be found in the Materials and Methods section). The model predicts a comparable relative biomass increase for discs with a channel width of 400 µm (congruent to the experiments, Fig. 3b). We further used the model to explore the influence of different channel widths (200–600 µm) and flow velocities (ranging from 11.5 µm s^−1^ to 1,150 µm s^−1^, Fig. S3). For all flow velocities and channel widths, we additionally explored intermediate angles compared to the physical experiments (15°, 45°, and 75°). However, we focus on the same angles (0°, 30°, 60°, and 90°) as in the experiments for visualization due to the predictable behavior of the intermediate angles (Fig. 3b). At higher bulk flow velocities (simulated as 1,150 µm s^−1^, or approximately 100 m d^−1^) even a small channel or pore (300 µm) with a pore flow velocity of approximately 50 µm s^−1^ can increase the total population growth by more than 12% (Table S2).

**Fig 4:**
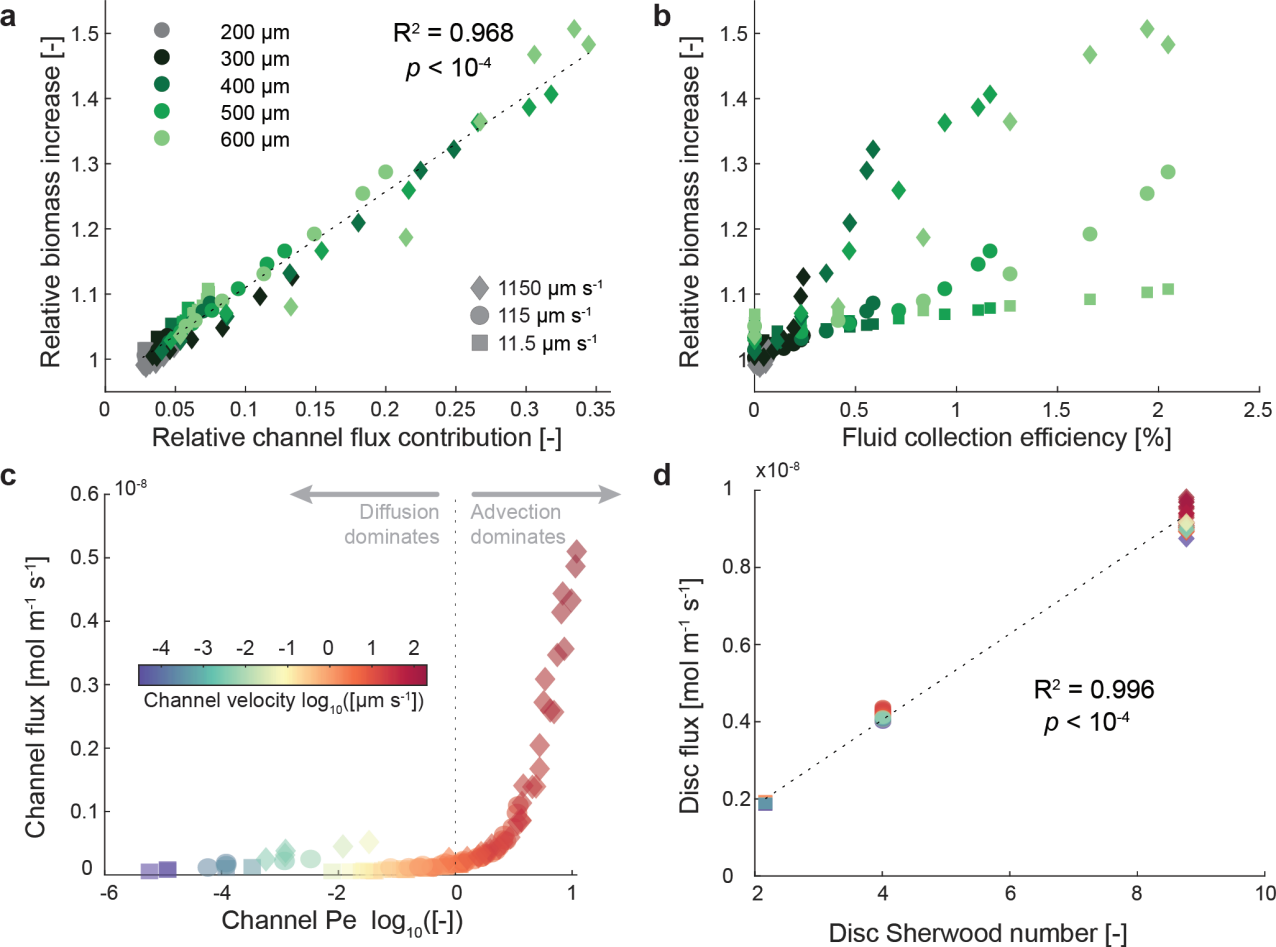
Contribution of channel and disc flux to the relative biomass increase. (**a**) Relationship between the relative channel flux contribution (percentage of the total flux through the channel wall) to the relative biomass increase. (**b**) Relationship between the fluid collection efficiency (the fraction of the flow passing through the disc from the flow approaching the disc) and the relative biomass increase. (**c**) Relationship between the channel Pe and the total flux through the channel. (**d**) Relationship between the disc Sherwood number and the total flux across the disc surface (neglecting flux through the channel).

We further explore the influence of different flow velocities and pore characteristics (channel angle and width) on the relative biomass increase by quantifying the total flux of the limiting substrate (in our case oxygen) to the discs in the simulations (Fig. 4). Oxygen flux via the channel is the main driver of the relative biomass increase (Fig. 4a). The relative channel flux contribution is the ratio of the oxygen flux through the channel walls to the total oxygen flux (i.e., the sum of flux from the channel and disc surface). For wide channels at high flow velocities, oxygen supply through the channel can contribute up to 35% of the total supply to the disc. Interestingly, this is below the relative contribution of the channel surface to the total surface (39% calculated based on geometry), suggesting that even at 600 µm channel width, the two halves do not yet behave like distinct discs from a nutrient supply perspective. Contrary to our initial expectation, the fluid collection efficiency (defined as the fraction of liquid passing through the disc from the total approaching liquid) is not a good predictor of the relative biomass increase (Fig. 4b). This is because the fraction of fluid passing through the channel from the total fluid approaching the disc does not change with bulk flow velocity and therefore does not reflect the elevated nutrient supply and relative biomass increase through the channel (Fig. S4). As shown in Fig. 4b, even though discs show very similar fluid collection efficiencies for larger channels at different bulk flow velocities (~2%), the relative biomass increase is vastly different, ranging from ~10% for fluid velocities of 11.5 µm s^−1^ to ~50% for fluid velocities of 1,150 µm s^−1^.

To disentangle the relative contribution by advective flows through the channels and increase nutrient flux due to flow velocity, we calculate the Pe associated with the channel. As shown in Fig. 4c, Pe is a good predictor of how advective fluxes enhance the total nutrient flux through the channel. Overall, nutrient flux through the channel is only significant at Pe > 1 where advective mass transport dominates (Fig. 4c). However, since Pe is a function of the fluid velocity inside the channel (in our case, driven by the bulk fluid velocity, angle of the channel with respect to the bulk fluid flow, and width of the channel) and channel width, different combinations of these parameters give rise to a similar regime when it comes to the relative importance of diffusive vs advective fluxes. For instance, channel widths of 200, 400, and 600 µm at angles of 75°, 75°, and 45° and bulk flow velocities of 1,150, 115, and 11.5 µm s^−1^ result in a very similar Pe of 0.8, 0.7, and 0.7, respectively. This illustrates how vastly different bulk flow regimes and microscale geometries give rise to comparable nutrient supply and growth conditions from a microbial perspective. In addition to the elevated nutrient supply through the channel, a higher bulk flow velocity also increases the nutrient flux across the disc’s hemispherical surface (Fig. 4d). This is due to a thinning in the diffusive boundary layer which is captured by the dimensionless Sherwood number (defined as the ratio of the total combined mass transport from diffusive and advective fluxes to diffusive mass transport alone) (25). For our system, we can calculate the Sherwood number from the cylinder diameter, bulk flow velocity, and nutrient diffusivity (25), which results in Sherwood numbers of 8.7, 4, and 2.2 for bulk flow velocities of 1,150, 115, and 11.5 µm s^−1^, respectively. These correlate well with the overall increase in nutrient flux through the hemispherical surface as shown in Fig. 4d. Importantly, a Sherwood number of 2.2 suggests that despite an overall low bulk flow velocity of 11.5 µm s^−1^, the additional advective flux due to flow around the cylinder more than doubles the overall nutrient supply to the resident microbial community even in the absence of any channel or pores through the cylinder.

### Microscale spatial patterns emerge from localized nutrient availability

**Fig 5:**
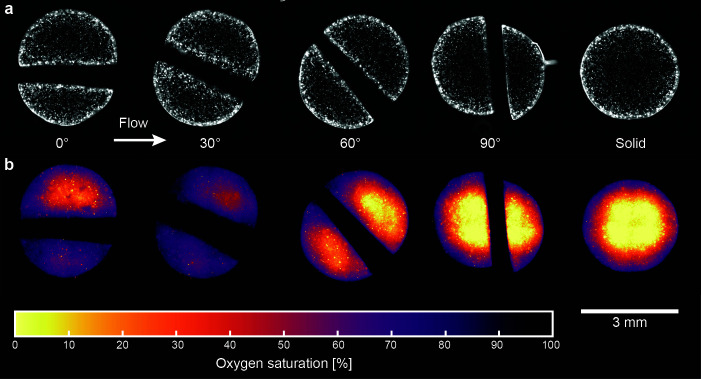
Biomass and oxygen distribution within the agarose discs. (**a**) Microbial biomass is visualized as brightfield images where brighter areas are indicative of higher biomass. Images were enhanced (rolling-ball background removal and brightness correction) to emphasize the biomass within the discs. (**b**) Spatial oxygen concentrations visualized using fluorescent nanoprobes.

In addition to affecting the overall microbial community in the discs, emerging nutrient gradients change the localized growth conditions at finer scales within the disc (Fig. 5a). For channels that support substantial flow (0° and 30°), the areas inside the disc adjacent to the channel walls are well-oxygenated and show higher biomass compared to the center of the semi-discs (Fig. 5b). In discs where the channel is at an angle of 60°, the anoxic areas expand within the discs. In addition, there is an asymmetry in the oxygen distribution along the channel, suggesting that even the fluid within the channel becomes devoid of oxygen toward the end of the channel. Finally, discs with a channel perpendicular to the flow where mass transport is dominated by diffusive fluxes (90°) show a spatial oxygen pattern congruent to a solid particle where regions directly adjacent to the channel remain anoxic.

**Fig 6:**
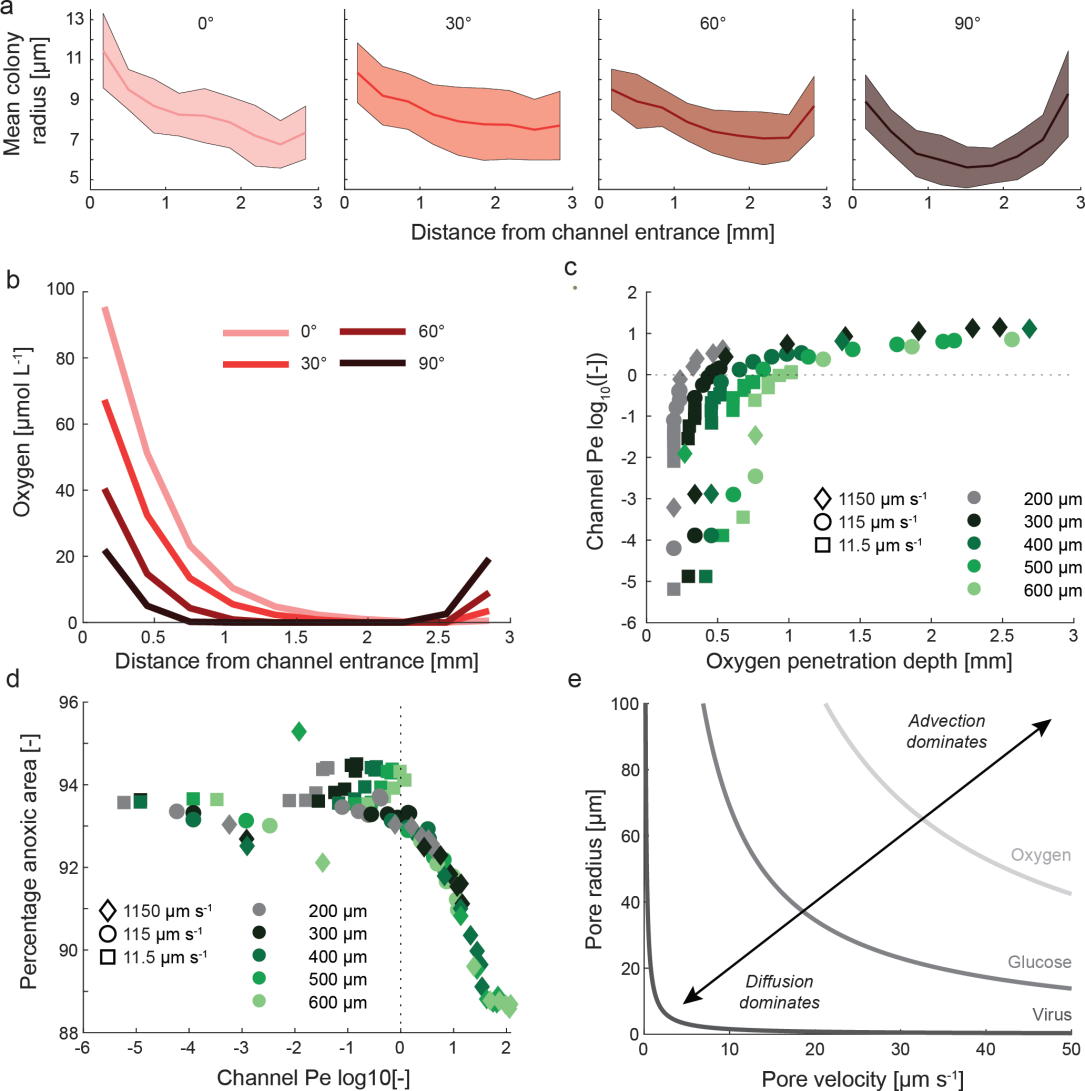
Microscale spatial patterns of colony size and oxygen distribution within the discs. (**a**) Colony size as a function of the distance to the channel entrance (channel entrance exposed to oncoming flow) for all channel angles in the experiments. Solid lines show the mean with the shaded area ± one std around the mean. (**b**) Oxygen concentration at the channel center as a function of the distance to the channel entrance for a flow velocity of 115 µm s^−1^ and 400 µm channel width. (**c**) Oxygen penetration depth (defined as the distance from the channel entrance when oxygen at the channel center drops below 1 µmol L^−1^) in relation to the channel Pe for all flow velocities, channel widths, and angles. Penetration depths that exceed 3 mm are omitted. (**d**) Percentage of the area that is anoxic (defined as the percentage of the area where oxygen concentrations are below 1 µmol L^−1^) in relation to the channel Pe for all flow velocities, channel widths, and angles. (**e**) Delineation between advective and diffusive dominated transport (curves indicate the threshold with Pe = 1) considering nutrients and particles with varying diffusivities.

The influence of advective oxygen contributions becomes apparent when observing the change in colony size as a function of the distance of the channel entrance. Here and throughout the manuscript, the distance from the channel entrance is the physical distance from the channel entrance that is exposed to the oncoming flow (windward). Bacterial colonies show a symmetrical pattern for the 90° angled channel with larger colonies at the channel periphery (0 and 3 mm) and smaller colonies at the center of the disc (distance from the channel entrance of ~1.5 mm, Fig. 6a). In this case, there is no flow through the channel, and the increase in colony size at the periphery is due to an increase in diffusive fluxes from the surrounding flow. With increasing flow through the channel from 60° to 0° (in line with the flow), this pattern becomes more skewed toward larger colonies directly at the channel entrance. This pattern is congruent to the increased oxygen availability supported by advective fluxes into the disc (Fig. 6b, Fig. S5). Channels perpendicular to the flow show a very rapid decline in oxygen concentration along the channel axis, whereas oxygen may penetrate deeply into the disc for channels in-line with the flow. The oxygen penetration depth (defined as the distance from the channel entrance when the oxygen concentration falls below 1 µmol L^−1^) is critically dependent on the flow within the channel (driven by the channel orientation and width) and the bulk flow velocity (see Table S3). One micromol per liter of oxygen was taken as a semi-arbitrary threshold where aerobic respiration becomes negligible (26). For the case of a channel perpendicular to flow, oxygen penetration is independent of the bulk fluid velocity (e.g., 450 µm into a 400 µm wide channel at all velocities), suggesting that oxygen penetration is solely from diffusion. In contrast, the penetration depth for a 400 µm channel at a 30° angle relative to the bulk flow direction increases from 450 to 750 µm to complete penetration (>3 mm) for flow velocities of 11.5, 115, and 1,150 µm s^−1^, respectively. For a high flow velocity of 1,150 µm s^−1^, even a narrower channel of 300 µm increases the oxygen penetration depth nearly eightfold from 340 µm (when the channel is perpendicular to the flow direction and restricted to diffusive penetration) to 2.5 mm when the channel is in line with the flow.

The rapid and non-linear increase of the penetration depth with flow velocity can be explained by the channel Pe number (Fig. 6c). At lower Pe (values below the dashed line in Fig. 6c), the channel width is an important factor driving the penetration depth since the total diffusive oxygen flux into a wide channel is greater when compared to a thin channel (proportional to the channel cross-sectional area). Advective fluxes on the other hand dominate at higher Pe where oxygen can penetrate the whole disc, especially at higher flow velocities and wide channels. Channels, where oxygen does not fall below 1 µmol L^−1^ (i.e., oxygen fully penetrates the channel), have a mean Pe of 50. Advective fluxes not only determine the increase in growth but also affect the potential metabolisms occurring within the microbial hotspots. Although most of the discs remain anoxic in all conditions, an increase in oxygen supply via advective fluxes reduces the percentage of the area within the disc that is anoxic (Fig. 6d). When diffusion dominates (Pe < 1, to the left of the dotted line in Fig. 6d), the percentage of the disc that experiences oxygen levels above 1 µmol L^−1^ is approximately 6%. Importantly, discs that are exposed to low bulk flow velocity show a larger anoxic area when compared to the general trend [below log_10_(Pe) of 0]. In these discs, oxygen consumption exceeds the supply via diffusion and renders even the edge of the disc completely anoxic, resulting in a larger fraction of the disc area being anoxic. With the onset of significant advective fluxes (Pe > 1, to the right of the dotted line in Fig. 6d), the oxic area rapidly increases to approximately 12%. Although this may represent a minor increase relative to the total disc area, it doubles the niche where microbial cells can maintain aerobic respiration.

Finally, advective fluxes not only enhance oxygen supply but also influence all mass transport to and from the disc. Using the Péclet number, we can compute the pore characteristics in which advective fluxes become significant for different entities (Fig. 6e). Here, the shape of the curves (which indicate Pe = 1) is purely a function of the entity’s diffusivity and separates pore characteristics where diffusion dominates (combinations of flow velocity and pore size below the curve) from those where advective fluxes dominate mass transport. For rapidly diffusing entities such as oxygen, diffusive fluxes dominate except for large pores with high flow velocities. For slower diffusing entities such as glucose or especially small particles such as viruses, small interstitial flow rates in narrow pores already contribute significantly to the overall transport of the entity through advective fluxes. As such, limitation by larger substrates can be greatly reduced, and viral infection rates greatly enhanced by the intra-particle advective flow. However, it is important to note that this analysis is only representative of macropores that permit advective flows. For gel matrices in natural microenvironments such as the cell clusters in biofilms, the microporous structure is typically too small (on the order of nanometers), and mass transport solely occurs via diffusion (24).

## DISCUSSION

Our results highlight the importance of interstitial advection for nutrient supply and the proliferation of bacteria in microenvironments that are exposed to bulk flow such as biofilms on riverbeds and intertidal zones, the periphyton, marine snow particles, or flocs in activated sludge. Within these microenvironments, microbial cells typically grow in a gelatinous matrix that permits diffusion via micropores. Discontinuities within this gel matrix (such as large pores or channels) permit advective flow through the matrix if pressure gradients across the discontinuities imposed by the surrounding fluids are sufficient to overcome the frictional resistance. This is an important distinction to microbial growth in porous media (such as soils, sediments, aquifers, or engineered systems like packed bed reactors or trickling filter systems in wastewater treatment) where numerical studies of the interaction between the biofilm surface roughness and mass transport (27) and simulations of coupled microbial growth and flow in porous media (28–32) have provided key insights on how flow can shape localized growth conditions. However, in contrast to the microenvironments we focus on in this study, microbial communities in porous media attach to the solid structures and create streamers that can clog macropores (33, 34) and alter preferential flow paths within the porous media with consequences for nutrient supply (35, 36). Thus, although similarities exist between the systems, caution needs to be exerted when extrapolating insights from our analysis to other porous media. In our system, we controlled the pore velocity (and associated advective nutrient supply) by changing the angle relative to the flow to achieve a wide range of conditions without changing the disc’s geometry. By doing so, we strategically disentangled the impact of elevated nutrient supply from interstitial advective fluxes through the channels (Fig. 4c) vs the disc’s surface related to the bulk flow velocity (Fig. 4d). In our system, a modest pore velocity of 10 µm s^−1^ supported an increase in carrying capacity of approximately 10%, where faster flow velocities inside channels support an increase in carrying capacity of up to 50% (Tables S2 and S4). Considering that we find these results for a rapidly diffusing substrate such as oxygen, advective fluxes may contribute a substantial fraction of the total nutrient supply to microenvironments for substrates with lower diffusivities such as larger organic molecules or even viruses (Fig. 6e).

The question remains if pores in microenvironments that support advective fluxes are widespread or if exopolymers produced by the resident microbial community will rapidly fill any voids within the microenvironments (37). Early studies on flow through biofilms (38) revealed a network of pores in between bacterial clusters of up to 50 µm radius that support high interstitial flows between 2,000 and 15,000 µm s^−1^. Similarly, visualizing the flow field around and through bacterial flocs (39) and marine aggregates (40) using particle image velocimetry has demonstrated the existence of flow penetrating through the particles, although similar experiments investigating the micro-hydrodynamics around marine snow particles did not show conclusive evidence supporting interstitial flow (24). However, in these latter experiments, the marine particles were impaled on a needle to visualize the flow which selected for more robust and potentially less porous particles. Natural marine particles also demonstrate high fluid collection efficiencies (i.e., the percentage of the approaching flow that passes through the particles) ranging from 12% to 40% for highly porous particles (40), which is an order of magnitude higher when compared to the collection efficiency calculated in our experiments (Fig. 4b) and suggests that the carrying capacity in the former may be elevated beyond our observations. Finally, it has been shown experimentally by comparing measured oxygen consumption rates and calculated oxygen supply from oxygen gradients that diffusion alone cannot supply sufficient oxygen to a marine particle to explain its high consumption (41). Within those experiments, additional interstitial advective fluxes between 5 and 40 µm s^−1^ were required to account for the discrepancy between observed oxygen consumption and that predicted solely by diffusive theory (41). Together, these experimental results suggest that large pores and pore networks through aggregates and within biofilms that permit advective fluxes are omnipresent features that greatly alter the localized growth conditions of microbial cells within the microenvironment.

Within an individual microenvironment, multiple gradients emerge rapidly as a function of bacterial nutrient consumption or intermediate metabolite production. In diffusion-dominated microenvironments, localized anoxia develops (42) that permits anoxic metabolisms such as denitrification (9, 13, 43) or sulfate reduction (10). Enhanced oxygen supply due to advective fluxes can change the landscape of metabolisms occurring within the microenvironment and restrict anoxic metabolisms to a fraction of the microenvironment when compared to a diffusion-dominated scenario. An oxygenated periphery can significantly reduce the anoxic niche in the presence of a channel that elevates oxygen supply when compared to a solid particle (Fig. 6d), especially when considering that nutrients supplied from the periphery are now intercepted in the oxygenated layer. Fundamentally, this is only relevant for large aggregates with high interstitial flows that support deep oxygen penetration into the channel via advective fluxes (Fig. 6c). In contrast, we observe a shallower oxygen penetration depth into the channel for most conditions. This presents an interesting scenario where advective fluxes do occur, but microbes keep pace (colonies grow larger), and oxygen is rapidly consumed such that the bulk of the aggregate remains anoxic. These advective fluxes may thus increase the supply of anaerobically respired nutrients such as nitrate or sulfate without disrupting the anoxic niche. By doing so, the permeation of the aggregates not only increases the total carrying capacity but additionally elevates anaerobic metabolism occurring within the microenvironment. This is not only the case for electron acceptors such as described above but for any nutrient supplied from the bulk liquid (Fig. 6e).

Advective fluxes may shift the limiting nutrient within a microbial hotspot, especially if there is a large discrepancy in diffusivity such as between oxygen and larger organic molecules. In a purely diffusion-dominated system, substrates with higher diffusivity will result in a higher overall substrate flux for the same cross-sectional area when compared to a substrate with lower diffusivity (assuming equal concentration and driving gradient). This generates a scenario where one substrate is more available simply due to the diffusive characteristics. In contrast, if advective fluxes dominate, they are supplied at their relative concentration in the bulk fluid, and the diffusive gradient is disrupted, which can shift the system to a different limiting nutrient depending on the uptake stoichiometry between the substrates. For example, advective fluxes in a carbon-limited system may elevate the carbon supply such that the system becomes oxygen limited since their diffusivities often differ significantly. Oxygen (44) has a diffusion coefficient of 18 × 10^−10^ m^2^ s^−1^, whereas L-valine (45), citrate (46), and sucrose (37) have diffusion coefficients of 6.6, 5.9, and 4.8 × 10^−10^ m^2^ s^−1^, respectively (or 2.7, 3, and 3.7 times lower than oxygen). Considering that the stoichiometry for aerobic respiration between oxygen and a six-carbon sugar such as citrate is 6:1, a small increase in carbon flux can rapidly deplete oxygen if its supply does not increase correspondingly. However, this process is not generalizable as it is a function of the substrate concentrations, their uptake stoichiometry, and diffusivity, among others.

Finally, advective fluxes may not only elevate nutrient supply, but they may also flush away intermediate metabolites from the microenvironments. For example, the release of nitrous oxide, both an intermediate metabolite in denitrification and a critical greenhouse gas (47), may result in a local loss of energy-yielding substrates but also modulate the interaction between these microenvironments and biogeochemical cycles. On the other hand, the removal of intermediate metabolites may also benefit the resident microbial communities in the case where these inhibit growth (17), a process that would be further enhanced by the permeation of the particle. Localized flow and the relative change in diffusive vs advective transport also modify the way organisms such as non-motile cells or viruses interact with microenvironments. Due to their large size and associated low diffusivities, interstitial fluxes on the order of a few microns per second already elevate the potential colonization and infection rate by non-motile cells and viruses, respectively (Fig. 6e). For example, porous marine snow that permits interstitial advective flows is colonized more efficiently by both motile and non-motile cells when compared to a rough or smooth surfaced particle (48). However, while motile cells showed an average of 10-fold increase in colonization for porous particles, this varied considerably for non-motile cells ranging from a negligible increase to an over 1,000-fold increase. This is because non-motile cells (and by analogy viruses) rely on a direct, stochastic encounter with the particle (where the microstructure mediates the flow in and around the particles), whereas motile cells simply benefit from a reduction in flow velocity and ability to colonize through random or chemotactically guided motion (48). Finally, the permeation of microenvironments also changes the way these interact with each other in terms of aggregation. Activated sludge in wastewater treatment plants and marine particles develops through direct collisions of smaller colloids supplemented by exopolymeric substances produced by microbes (49, 50). Similarly, biofilms in streams or intertidal zones can import colloids as evidenced by the rapid incorporation of metal nanoparticles (51). The presence of macropores that promote interstitial flows can shift the dynamics of these processes. Large particles that are typically more porous scavenge smaller particles at rates that rapidly exceed theoretical collision efficiencies in the absence of particle permeation (52).

Our results demonstrate the importance of microscale variations in flow for the development and functioning of microbial communities within microenvironments due to a change in nutrient supply. Indeed, the overall supply to microbial hotspots is a delicate balance between advective and diffusive nutrient fluxes where their relative importance is captured by the dimensionless Pe number. Elevated nutrient fluxes due to supply through pore spaces directly impact the carrying capacity of the microbial hotspots (Fig. 4a) and are tightly linked to the Pe (Fig. 4c). The increase in nutrient supply due to dominant advective fluxes defined as Pe ≥ 1 also delineates the threshold where oxygen penetration into the channel or pore becomes significant (Fig. 6c) and alters the fraction of the hotspot where aerobic growth may occur (Fig. 6d). Importantly, the Pe can be calculated directly from the geometry of the pore spaces, localized fluid velocity, and diffusivity of the nutrient under consideration. From this perspective, characterizing the localized flow regime and microscale geometry of microbial hotspots can explain potential shifts in microbial metabolism within microbial hotspots but also enable the informed design for engineering applications.

In summary, the elevated mass transport from advection on top of diffusion not only impacts the carrying capacity of microbial hotspots but also shapes the emerging niches within the microenvironments with consequences for the spatial occurrence of diverse metabolisms, the proliferation of specialized species, and the overall diversity of the resident community. These changes in microbial communities greatly impact metabolisms associated with global biogeochemical cycles and overall ecosystem function. Furthermore, advective fluxes change the colonization or detachment of non-motile bacteria from these microenvironments or critically facilitate the contact between microbial hotspots. However, how the permeation of microenvironments and associated changes to the local biogeochemistry and ecology of microbial hotspots collectively impact the functioning of meso- and global-scale processes in the Earth system remains unknown.

## MATERIALS AND METHODS

### Bacterial strains and culture conditions

We used a fluorescently tagged *Pseudomonas aeruginosa* PA14 that constitutively expresses a yellow fluorescent protein for all experiments (48). We used Lennox lysogeny broth (5 g L^−1^ NaCl, BD Life Sciences, further abbreviated LB) media for overnight cultures and in the experiment and supplemented the overnight cultures with 300 µg mL^−1^ Trimethoprim (Trp) to avoid contamination.

### Fabrication of millifluidic devices

We adapted a previously described experimental system that mimics a settling marine particle amenable to microscopy (13). We fabricated the millifluidic devices using two 1 mm thick microscope slides (VWR, Vistavision Microscope Slide, 75 × 25 × 1 mm) with red silicone sheets (Diversified Silicone Products, #5038GP-032, 50 Duro, 1/32 in thick; McMaster-Carr #1460N21) in between the glass slides. The flow chamber contains five 8 mm diameter circles spaced 10 mm apart that are connected by a 5 mm wide channel (Fig. 1). The silicon sheet is bonded to the bottom glass microscope slide using plasma corona treatment. To visualize and quantify the growth of bacterial colonies in response to interstitial advective fluxes, we created hydrogel discs in the shape of a disc with a 3 mm diameter and 1 mm height and a single 400 µm channel through the particles where we control the advective flux through the particle by changing the angle of the channel relative to the fluid flow. We prepared the hydrogel discs using low-melting agarose as previously described (13). In brief, we dissolved 15 mg of low melting agarose (Agarose low gelling temperature, Sigma-Aldrich, CAS# 39346-81-1) in 1 mL of LB medium in a block heater at 70 °C. We then cooled the agarose directly in the block heater to 40 °C. We then added 10 µL of a bacterial suspension containing 10^7^ cells mL^−1^ (final bacterial concentration in the agar of 10^5^ cells mL^−1^) to the liquid agarose and vortexed rigorously to ensure that the inoculated bacterial cells are homogeneously distributed within the agarose. We then create a 1 mm thick sheet of agarose using a 3 mL syringe (BD, 3 mL Luer lock) and a blunt needle (Industrial Dispensing Supplies, 22G) by dispensing the molten agarose in between two microscope slides that are separated by 1 mm using a separate microscope slide. Once solidified (15 minutes at room temperature, ~20 °C), we punched the agarose discs from the agarose slab using a 3 mm diameter biopsy punch (Integra Miltex, Disposable Biopsy Punch, 3 mm) and bisected the particles into equal halves using an X-ACTO knife. We then transferred the two halves into the flow chamber and manually positioned the two halves at approximately 400 µm apart with the required angle relative to the fluid flow. Finally, we placed the microscope cover glass containing the inlet and outlet ports on top of the assembly, sealed the whole experimental system by applying pressure to the silicone layer, and reinforced the seal with labeling tape.

### Millifluidic growth conditions and image acquisition

We controlled the flow within the millifluidic device using a syringe pump (Harvard Apparatus, PHD ULTRA). We aligned five discs in each millifluidic device with their internal channels between 0° (inline) and 90° (perpendicular) to the flow to control the relative contribution of advective and diffusive fluxes to the overall mass transport without changing the geometry of the microenvironment. We then imposed a settling velocity of 10 m d^−1^ around the particles (LB media) for 24 h. After this time, we visualize the bacterial colonies within the particles using a Nikon Ti2-E inverted microscope equipped with an Andor Zyla 4.2 sCMOS camera. The microscope was controlled using Nikon Elements (v.4), and all images were captured in 16-bit with a 10× objective (Plan Fluor DLL). Each hydrogel disc was visualized at five depths 50 µm apart, and the center was located approximately at the middle of the channel thickness (i.e., ~500 µm from the bottom glass slide which we located using the perfect focus system of the Nikon Ti2 microscope). Each depth layer was captured as a 4 × 4 tile scan with a 20% overlap that was automatically stitched by the Nikon Elements software. Individual images of the tile scans were captured at an Illuminator Iris intensity of 30 and shutter speed of 50 ms for all particles to ensure equal illumination of each particle and thus reliably quantify the colonized population. These settings resulted in an optimal dynamic range within the images for particles and thus required the least amount of post-processing.

### Digital image analysis to extract the bacterial colony size

We analyzed the images using MATLAB (R2020) using the image analysis toolbox. Overall, we initially classify if the image contains a solid disc (no channel), contains two semi-discs (and therefore a channel), or if the discs have moved (in which case we do not analyze the image). We classify discs based on the presence or absence of a channel using a custom algorithm. This algorithm employed a shape index filter to classify discs based on the measured circularity and elongation shape factor. The latter corresponds to the square root of the ratio of the second moments of an object around its principal axes (53). Discs that deviated from the expected shape of intact discs were then either classified as semi-discs or discarded if did not meet semi-disc characteristics (e.g., semi-discs that moved during incubation). For the discs matching the geometric characteristics of semi-discs, they were further processed to extract relevant features of the channel. We created a mask to extract the channel itself using a black hat transform (differences between the closed image and the image itself) following a white hat transform (difference between the input image and its opening). These transformations were required to identify the channel area between the semi-discs. We subsequently utilized this channel to measure the exact angle orientation, width, and length of the channel. Finally, we segmented the colonies, and their spatial positions relative to the channel and particle edges were obtained using methods described elsewhere (7, 13).

### Quantification of bacterial growth

We use a solid disc (i.e., without a channel) as a control where mass transport is limited to diffusion through the microporous agarose gel matrix and does not permit any interstitial flows. We determine the influence of interstitial flow on the carrying capacity by examining the pattern of colony size as a function of the radial distance from the nutrient-rich periphery in solid discs and ascribe large colonies that diverge from this pattern in discs containing a channel to advective fluxes. We quantify the increase in biomass due to advection as the ratio of the total observed biomass within those discs (the sum of the total area identified as colonies within an image) to a hypothetical scenario where we omit the anomalous colonies (for more details, see Fig. S1 and S2). For each disc, this approach is calculated for five depths inside the agarose discs (at heights of 200, 300, 400, 500, and 600 µm from the bottom glass slide). Since bacterial cells are homogeneously distributed within the agarose, this represents an average estimate of the relative biomass increase across the whole disc. The biomass fold increases calculated here and reported throughout the rest of the manuscript thus represent the increase in biomass due to advective nutrient contributions relative to a solid disc. We use this approach because it effectively normalizes the biological variance between replicates and isolates the influence of elevated nutrient supply due to advective fluxes (Fig. 3a).

### Statistical analysis

In total, we performed eight replicates of the experiment. Within the millifluidic devices, the sequence of channel angles through the discs was arranged randomly to exclude any potential bias due to the position inside the device. Any image where the automatic algorithm failed to determine the edge of the disc (e.g., due to an air bubble proximal to the disc or two semi-discs that shifted and merged) was excluded from the analysis. For the in-line particles, this resulted in the omission of three discs and thus a total replicate number of 5. Before any statistical test, data were tested for homoscedasticity using Bartlett’s test and normality using the Anderson-Darling test. Due to differences in variance between groups, a comparison of the relative biomass increases between the different channel angles (Fig. 3a) was performed using a Welch alternative ANOVA. We further used a linear model to assess the relation between the relative biomass increase and the channel nutrient flux (Fig. 4a) and the disc surface flux with the disc Sherwood number (Fig. 4d).

### Visualization of the flow field using particle image velocimetry

We used fluorescent microspheres embedded in the flowing media to visualize and quantify the flow around the discs using PIV. We created microfluidic devices made from polydimethylsiloxane each containing a disc with a 400 µm channel at defined angles (0°, 45°, and 90°). Negatives of the devices were 3d printed using a Form3 SLA printer (Formlabs, MA, V4 clear resin, 25 µm resolution). The dimensions of the flow chamber are congruent to the experimental system. We diluted fluorescein isothiocyanate-marked (FITC) microspheres (Sigma-Aldrich, 90305–5ML-F, 1 µm diameter, excitation 490 nm, emission 525 nm, and 2.5% solids concentration) 2,000× in ultrapure reverse osmosis (milliQ) water which we flowed through the devices at 115 µm s^−1^ to imitate experimental conditions. We then acquired 20 images at 50 ms intervals (maximum microsphere displacement of 5.75 µm or 9 pixels using the same plan fluor 10× objective) to capture the movement of individual microspheres. We covered the whole disc by composing a tile scan (six by four images). We applied a background correction algorithm to remove any signal from reflections within the chip (subtracting the median of all 20 images) and a histogram equalization before stitching the images for each time point geometrically. The product of these operations is 20 images covering the whole disc which we analyzed using PIVlab (54). We used standard parameters suggested in PIVlab (version 2.63, CLAHE enabled, 64 px interrogation area and 32 px step for pass 1, 32 px interrogation area, and 16 px step for pass 2) for the analysis, a custom mask to exclude the disc area, and calibration based on the pixel size (0.65 µm per pixel) and image acquisition frequency (50 ms) to convert the resulting normalized velocity fields to flow fields.

### Quantification of the spatial oxygen concentration using fluorescent nanoprobes

We used oxygen nanoprobes (Oxnano, PyroScience, Germany) to visualize and quantify the spatial oxygen concentrations in the agarose discs. We added nanoprobes at a final concentration of 50 µg mL^−1^ to the molten agarose before gel-casting the agarose slab. We visualized the fluorescence of the oxygen-sensitive nanoprobes using a Cy5 filter cube (excitation: 590–650 nm and emission: 662–737 nm) in 1-h intervals for 24 h. For each disc, we related the fluorescent intensity to the oxygen saturation using a two-point calibration (7, 13). Oxygen-rich conditions were obtained from the initial image (T0, before bacterial growth). For anoxic conditions, we exchanged the liquid media after the 24-h incubation period with the same media but supersaturated with sodium sulfite (10 g L^−1^).

### Digital twin simulations using the COMSOL simulation software

We used COMSOL Multiphysics v5.6 to create digital twin simulations of the experimental system to observe the spatiotemporal evolution of oxygen and associated bacterial patterns. The simulated geometry and equations used for describing bacterial growth are outlined below.

### Simulation geometry

We created a 2D time-dependent model that contains both the discs and surrounding flow channel (Fig. 1). We used a digital twin approach (i.e., 3 mm diameter particle inside 8 mm diameter chambers that is connected via 5 mm wide channels) and assigned a no-slip boundaries to the flow chamber and particle surface. The numerical grid used for the simulations (finite element method) is automatically created by COMSOL using an adaptive mesh algorithm with a grid size of ~20 µm adjacent to any boundaries (both the discs and the channel edges) increasing to a maximum of ~1 mm. Inside the discs, we include channels ranging from 200 µm to 600 µm that are rotated from 0° to 90° in 15° increments with respect to the flow. Due to our inability to precisely control the channel width and rotation in the millifluidic experiments, we explore the influence of these two parameters beyond the experimental geometry using the model. We simulate fluid flow inside the flow chamber by imposing a mean flow velocity across the entire device (11.5, 115, and 1,150 µm s^−1^) which generates flow inside the channel due to the geometry and resulting pressure distribution. In addition to flow through the channels, hydrogels permit flow through the hydrogel matrix due to their nanoporous structure which we can calculate for solid discs as a function of their size and permeability (52). However, due to the low permeability of hydrogels [e.g., 450 nm^2^ for 3% agarose hydrogel (55)], the fluid collection efficiency is merely 10^−7^ % and negligible when compared to the fluid collection efficiency driven by flow through the channels (>10^−2^ % for the majority of conditions, Fig. 4B). In addition, since the majority of this flow through the hydrogel is confined to a thin boundary layer (52) (in our case, ~50 nm assuming a linear relationship between agarose concentration and permeability), elevated nutrient supply due to flow through the hydrogel matrix only affects regions where diffusive fluxes dominate (characteristic time of diffusion for oxygen across 50 nm is on the order of 1 µs) and thus do not significantly alter nutrient fluxes. For this reason, we simulated the discs as impermeable solids in COMSOL where nutrient fluxes inside the discs are limited to diffusion. We used the COMSOL standard diffusion coefficient for oxygen in the water and agarose of 1.0 × 10^−9^ m^2^ s^−1^ and assigned a uniform initial cell density of 10^5^ cells mL^−1^ inside the disc with negligible diffusion of the cells to simulate immobilized cells. We set the oxygen concentration at the inflow and boundaries of the flow chamber (silicone) as fully saturated (0.258 mol m^−3^). Finally, the combined advection-diffusion-reaction system is solved in COMSOL using Eq. 2 and Eq. 3, where *N*_*ox*_ is the two-dimensional oxygen flux vector [mol m^−2^ s^−1^], *D*_*ox*_ is the oxygen diffusion coefficient [m^2^ s^−1^], *C*_*ox*_ is the oxygen concentration [mol m^−3^], *u* is the two-dimensional velocity vector [m s^−1^], and *v*_*ox*_ is the oxygen consumption rate [mol m^−3^ s^−1^]. The flow velocity field is calculated by solving the Navier-Stokes equation assuming Stokes flow (low Reynolds number regime).


Eq. 2 $$N_{ox}=-D_{ox}\nabla C_{ox}+C_{ox}u$$

Eq. 3 $$\frac{dC_{ox}}{dt}+\nabla N_{ox}=v_{ox}$$

### Bacterial growth and oxygen consumption

Bacterial growth was simulated using a population-based approach with a Monod-type oxygen limitation term to connect the underlying oxygen distributions to the emerging bacterial growth patterns. Since the population-based model is spatially explicit, it dynamically captures oxygen gradients emerging within the discs, which reciprocally alter the spatial distribution of growth rates within the discs. We additionally limited bacterial growth using a carrying capacity term to avoid physically impossible bacterial densities (exceeding space-filling conditions). We simulated the bacterial cells as a concentration of cells and represented the oxygen consumption and bacterial growth as reaction terms as shown in Eq. 4 and Eq. 5.


Eq. 4 $$\frac{dB}{dt}=\mu_{max}\frac{C_{ox}}{K_{ox}+C_{ox}}\left(1-\frac{B}{B_{max}}\right)B$$

Eq. 5 $$v_{ox}=Y\frac{dB}{dt}$$

Here, *B* is the concentration of cells [cells m^−3^], *µ*_*max*_ is the maximum growth rate [s^−1^], *K*_*ox*_ is the oxygen half-saturation coefficient [mol m^−3^], *B*_*max*_ is the carrying capacity that translates to the whole space filled by 1 µm^3^ bacterial cells [10^18^ cells m^−3^], *v*_*ox*_ is the oxygen consumption rate [mol m^−3^ s^−1^], and *Y* the oxygen demand [mol cell^−1^]. Values for all parameters are shown in Table 1. In addition, we ensured non-negative oxygen and cell densities for solver stability. The total simulated time was 24 h with a 2.4-second time-step. The state of the model at 24 h, corresponding to the experimental millifluidic device at the time of microscopy, was used for calculations and subsequent analysis.

**TABLE 1:** Parameters used for all variables in the COMSOL simulations

Parameter	Value	Units	Description	Reference
*µ*_max_	3.85 × 10^−4^	s^−1^	Maximum growth rate (simulated doubling time of 30 min)	(56)
*K*_ox_	6.3 × 10^−3^	mol m^−3^	Half-saturation coefficient for oxygen	(57)
*B*_0_	10^11^	cells m^−3^	Initial cell concentration (10^5^ cells mL^−1^)	
*B*_max_	10^18^	cells m^−3^	Carrying capacity (1 cell µm^−3^)	
*Y*	2.29 × 10^−17^	mol cell^−1^	Oxygen demand	(58)

## Data Availability

All extracted data and the underlying code for this study are available in Zenodo and can be accessed via this DOI: 10.5281/zenodo.8338298.

## SUPPLEMENTAL MATERIAL

Supplemental Information msphere.00185-24-s0001.pdf Supplemental tables and figures.

ASM does not own the copyrights to Supplemental Material that may be linked to, or accessed through, an article. The authors have granted ASM a non-exclusive, world-wide license to publish the Supplemental Material files. Please contact the corresponding author directly for reuse.
