# Supplementary material for: Microscale advection governs microbial growth and oxygen consumption in macroporous aggregates
Source: mSphere. 2024 Mar 26;9(4):e00185-24. doi: 10.1128/msphere.00185-24 (PMC11036798; doi:10.1128/msphere.00185-24)
Supplement: Supplemental Information — Supplemental tables and figures. [file msphere.00185-24-s0001.pdf]

Supplementary information to:

Microscale advection governs microbial growth and oxygen consumption in macroporous aggregates

Rachel Shen[1], Benedict Borer[1]*, Davide Ciccarese[1], Mehdi Salek[2] and Andrew R. Babbin[1]*

1: Earth, Atmospheric and Planetary Sciences Department, Massachusetts Institute of Technology, Cambridge, MA 02139, USA.
2: Department of Biological Engineering, Massachusetts Institute of Technology, Cambridge, MA 02139, USA.

Corresponding authors: Benedict Borer (bborer@mit.edu) and Andrew Babbin (babbin@mit.edu)

This PDF includes:

- SI Figures 1 – 4
- SI Tables 1 – 4

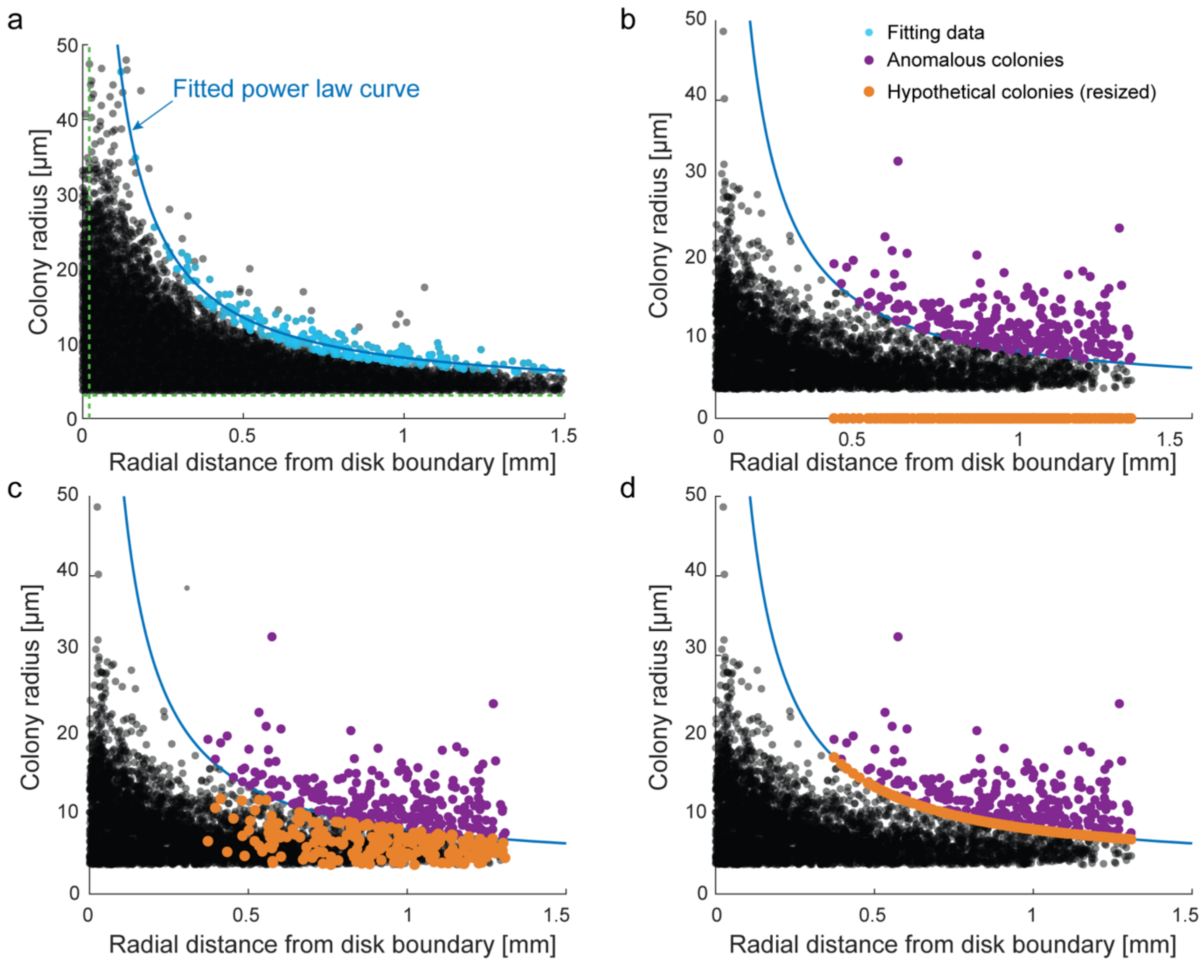

Supplementary Figure 1: Visualization of the approach used to determine the relative biomass increase. a) We fitted a modified power law equation (see methods section) to the envelope of the colony size versus distance from the disc boundary (fitting data shown in blue). We obtained the fitting data by using the same modified power law equation with initial estimates based on physical properties from the experimental system (an out-of-focus band at the disc boundary of 50 µm for the x-axis shift and minimum colony size for the y-axis shift, both denoted by dotted green lines) and selected colonies that deviate less than 2 µm from this hypothetical line. We then refitted the modified power law to the fitting data to obtain the final curve which we used to determine anomalous colonies in discs with a channel. We used three approaches to determine the relative biomass increase due to the presence of advective fluxes by calculating the ratio of the total observed biomass (including the anomalous colonies) and hypothetical biomass by omitting the anomalous colonies (**b**), assigning a colony radius between the minimum radius and fitted curve (**c**) or assigning a radius equal to the fitted curve (**d**).

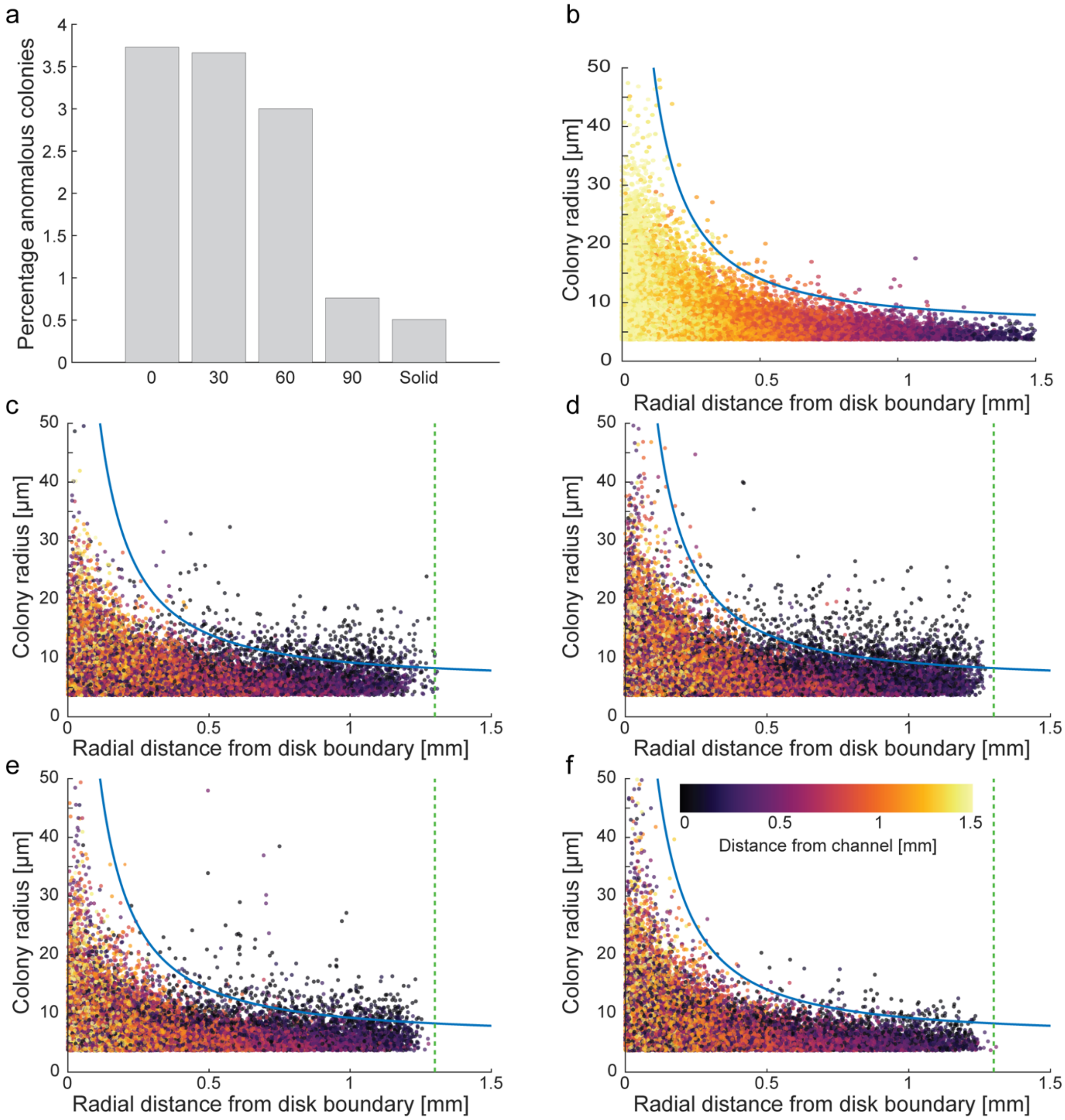

Supplementary Figure 2: Quantification and visualization of anomalous colonies in discs with a channel. a) Mean percentage of anomalous colonies identified in discs with different channel angles. **b)** Colony radius versus the radial distance from the boundary for the solid discs. Since these discs do not have a channel, the color indicates the distance from the disc center. Colony radius versus the distance from the boundary for discs with a channel angle of 0° (**c**), channel angle of 30° (**d**), channel angle of 60° (**e**), and channel angle of 90° (**f**). Typically, anomalous colonies (colonies above the blue threshold) are far from the disc boundary but close to the channel as indicated by the color scheme.

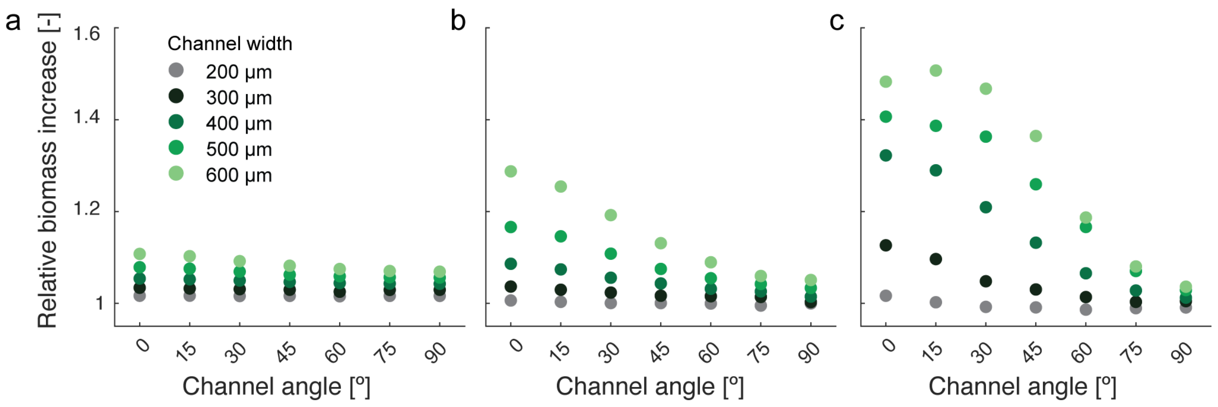

Supplementary Figure 3: Relative biomass increase as a function of flow velocity. We use COMSOL to characterize the influence of the flow velocity on the relative biomass increase by simulating a flow velocity of 11.5 µm s^{-1} (**a**), congruent flow velocity of 115 µm s^{-1} (**b**) or increasing to 1,150 µm s^{-1} (**c**). The data underlying this figure is provided in Supplementary Table 2.

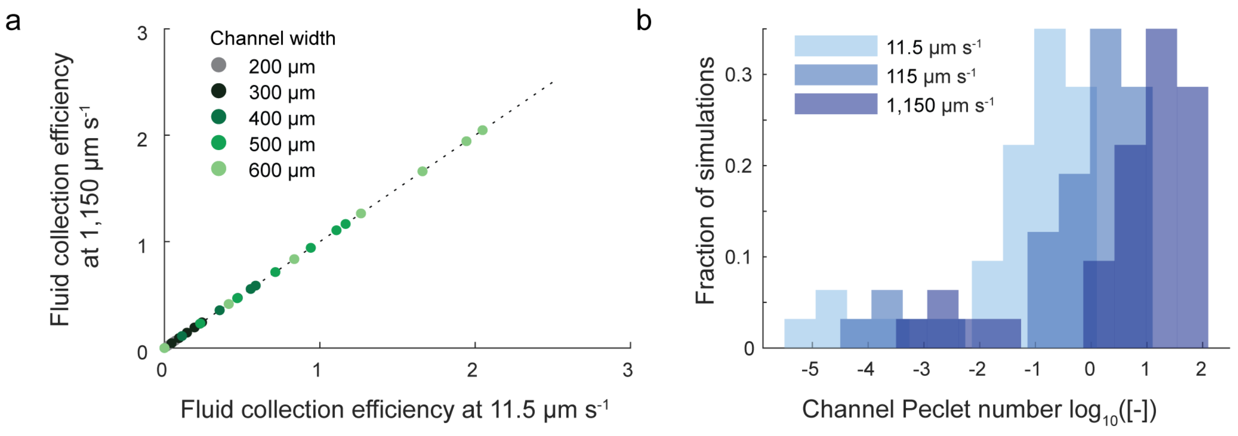

Supplementary Figure 4: Influence of flow velocity on the fluid collection efficiency and channel Pe. a) The fluid collection efficiency is not affected by the flow velocity as shown between the comparison at 11.5 µm s^{-1} flow velocity and 1,150 µm s^{-1} flow velocity. **b**) The flow velocity shifts the magnitude of Pe associated with the flow and geometry of the channel, but not the pattern associated with different geometries.

Supplementary Table 1: Relative biomass increase for all channel angles depending on the analysis algorithm. Relative biomass increase is calculated by omitting large colonies, stochastically reducing large colonies to a size between 0 and the upper limit of the fitted power law and reducing to the characteristic size determined by the fitted power law curve. Differences in the analysis procedure are highlighted in Supplementary Fig. 2. Values are given as mean ± *SD*.

	**0°**	**30°**	**60°**	**90°**	**Solid**
**Omitted**	7.52% ± 4.08%	7.39% ± 4.55%	5.82% ± 2.96%	1.18% ± 1.33%	1.38% ± 1.45%
**Stochastic**	5.16% ± 2.90%	5.25% ± 3.32%	4.09% ± 2.24%	0.73% ± 0.85%	0.80% ± 0.85%
**Reduced**	2.93% ± 1.85%	3.07% ± 2.10%	2.33% ± 1.39%	0.33% ± 0.42%	0.34% ± 0.38%

Supplementary Table 2: Percentage biomass increase for all channel angles and widths depending on the bulk flow velocity. We attribute negative values of relative biomass increase at medium and high flow velocities to small deviations in the flow field and numerical error.

Channel width (μm)	Bulk velocity (μm s^{-1})	Channel angle						
		0°	15°	30°	45°	60°	75°	90°
200	11.5	1.6	1.7	1.6	1.6	1.5	1.6	1.6
	115	0.6	0.3	0.1	0.1	0	-0.5	0
	1,150	1.7	0.2	-0.8	-0.9	-1.4	-1	-1
300	11.5	3.4	3.2	3.1	3	2.5	2.9	3
	115	3.7	3	2.3	1.7	1.5	1.4	0.3
	1,150	12.6	9.6	4.8	3	1.4	0.3	0.5
400	11.5	5.4	5.2	5	4.7	4.4	4.3	4.3
	115	8.6	7.4	5.6	4.3	3.2	2.6	1.5
	1,150	32.2	29	21	13.2	6.6	2.8	1.2
500	11.5	7.9	7.6	6.9	6.2	5.9	5.7	5.6
	115	16.6	14.6	10.8	7.5	5.5	4.2	3.3
	1,150	40.7	38.7	36.3	25.9	16.6	7	2.9
600	11.5	10.8	10.3	9.2	8.2	7.5	7	6.9
	115	28.8	25.4	19.2	13.1	8.9	6	5.1
	1,150	48.3	50.7	46.8	36.5	18.7	8	3.6

Supplementary Table 3: Maximum oxygen penetration for all channel angles and widths depending on the bulk flow velocity. Oxygen penetration is given in percentage of the channel length (3 mm).

Channel width (μm)	Bulk velocity (μm s^{-1})	Channel angle						
		0°	15°	30°	45°	60°	75°	90°
200	11.5	6	6	6	6	6	6	6
	115	8	8	8	8	7	6	6
	1,150	18	18	16	12	11	8	6
300	11.5	11	11	11	11	10	10	10
	115	17	17	15	14	13	11	11
	1,150	83	76	64	47	33	19	11
400	11.5	17	17	16	15	15	15	14
	115	36	33	29	25	22	17	15
	1,150	100	100	100	100	90	46	15
500	11.5	25	25	23	23	20	20	18
	115	72	69	59	48	38	27	20
	1,150	100	100	100	100	100	100	20
600	11.5	34	34	31	28	28	25	23
	115	100	100	100	86	62	41	26
	1,150	100	100	100	100	100	100	26

Supplementary Table 4: Maximum channel velocity for all channel angles and widths depending on the bulk flow velocity. Pore velocities are extracted from the simulations at the center of the pore (i.e., equidistant from both the channel walls and entrances).

Channel width (μm)	Bulk velocity (μm s^{-1})	Channel angle						
		0°	15°	30°	45°	60°	75°	90°
200	11.5	0.2	0.2	0.2	0.1	0.1	0	0
	115	2.1	2	1.3	1.2	0.8	0.4	0
	1,150	21.1	20	16.9	12.6	8.1	4	0
300	11.5	0.5	0.5	0.4	0.3	0.2	0.1	0
	115	4.9	4.6	3.9	2.9	1.9	0.9	0
	1,150	48.7	46.1	38.9	29.1	18.9	9.3	0
400	11.5	0.7	0.8	0.7	0.5	0.3	0.2	0
	115	8.6	8.2	7	5.2	3.4	1.7	0
	1,150	86.4	81.7	69.7	52.4	33.9	16.8	0
500	11.5	1.4	1.3	1.1	0.8	0.6	0.3	0
	115	13.9	13.1	11.1	8.4	5.5	2.7	0
	1,150	138.1	131	111.4	84.3	55.4	27.3	0
600	11.5	2	1.9	1.6	1.2	0.8	0.4	0
	115	20	19	16.2	12.3	8.2	4	0
	1,150	199.7	190.1	161.9	122.8	81.7	40.2	0
